# The Relationship Between Hospitalization Frequency of Acute Exacerbation of Chronic Obstructive Pulmonary Disease and Anxiety and Depression

**DOI:** 10.3389/fgene.2022.817727

**Published:** 2022-04-08

**Authors:** Ding Wen-tao, Chen Xue-xiu, Chen Zun-jiang, Chen Wei, Pan Cheng-feng, Fan Xing-ken

**Affiliations:** Department of Emergency, The Affiliated Cangnan Hospital of Wenzhou Medical University, Zhejiang, China

**Keywords:** COPD, frequency of hospitalization, anxiety, depression, hospitalization

## Abstract

Depression and anxiety are common in patients with COPD (chronic obstructive pulmonary disease), and anxiety and depression can increase the risk of hospitalization and the acute exacerbation of chronic obstructive pulmonary disease. The relationship between the frequency of hospitalization for acute exacerbation of COPD (AECOPD) and the anxiety and depression of patients is not fully understood. This study aims to analyze the relationship between the frequency of hospitalizations and anxiety and depression of patients of acute exacerbation of chronic obstructive pulmonary disease (AECOPD). A collection of 309 AECOPD patients admitted to the emergency department in our hospital from 2019 to 2020 were divided into anxiety group A and depression group D according to the Hospital Anxiety and Depression Scale (HADS) score and divided into A1 and D1 negative groups (≤7 Score), A2 and D2 suspicious groups (8–10 points), A3 and D3 confirmed groups (≥11 points) for paired analysis of anxiety and depression correlation and difference and comparison of the frequency of hospitalization in each group within 2 years. The results found that anxiety and depression were significantly positively correlated (r = 0.654, *p* = 0.000). Intra-group comparison shows that the difference between the anxiety-diagnosed and non-diagnosed groups and the depression subgroups are statistically *p* < 0.05; the comparison between the anxiety subgroup and the depression subgroup showed that there was a statistical difference between the confirmed group and the non-diagnosed group (*p* < 0.01). In short, AECOPD anxiety is positively correlated with depression, and depression is affected by the frequency of hospitalization earlier and gradually, and anxiety should be prioritized in the acute phase.

## Introduction

Chronic obstructive pulmonary disease (COPD) is a systemic disease, where the airflow limitation is not completely reversible and develops progressively, and it has the characteristics of repeated aggravation and progressive deterioration, which seriously affects the patient’s health. Its complications include cardiovascular disease, skeletal muscle dysfunction, metabolic syndrome, osteoporosis, depression, anxiety, lung cancer, etc. (Li et al., 2019; Wang and Jiang, 2020; Zhang, 2021). Anxiety and depressive disorder are two of the common complications of COPD patients, but they are also the most neglected complications. They can lead to a decrease in the desire to seek medical attention and reduced compliance in COPD patients. At the same time, anxiety and depression can be used as independent risk factors to affect the acute exacerbation of chronic obstructive pulmonary disease, the frequency and length of hospitalization, and seriously affect the quality of life of COPD patients (Xu et al., 2010; Song, 2017; Soler et al., 2021). At present, the increase in hospitalization frequency and the influence of anxiety and depression in AECOPD patients are rarely reported. Data analysis of this research group found that the increase in hospitalization frequency of AECOPD will have varying degrees of impact on anxiety and depression, causing a vicious circle.

## Materials and Methods

### Research Object

The study subjects selected 309 patients with AECOPD admitted to the emergency department from January 2019 to December 2020. Inclusion criteria were as follows: the first diagnosis met the AECOPD diagnostic criteria, and the patients signed the informed consent form to complete the questionnaire. Exclusion criteria were as follows: ① the first diagnosis is not AECOPD; ② serious condition, impaired consciousness, abnormal thinking, etc. combine to not allow the patient to complete the questionnaire; ③ combined with neuropsychiatric and other serious system diseases. This study has been approved by the hospital ethics committee (EA:K201901012), and informed consent was obtained from all patients.

### Data Collection and Group Comparison

We collected each patient’s gender, age, and body mass index (BMI), checked previous visits and counted the number of hospitalizations within 2 years, used the certified Chinese version of the Hospital Anxiety and Depression Scale (HADS) to score anxiety A and depression D, and divided them into anxiety according to the reference value. Each subgroup of group A is as follows: A1 (≤7 points) is the negative group, A2 (8–10 points) the suspicious group, and A3 (≥11 points) the confirmed group; each subgroup of depression group D is as follows: D1 (≤7 points) is the negative group, D2 (8–10 points) the suspicious group, and D3 (≥11 points) confirmed group. The differences in the frequency of hospitalizations within 2 years were compared within each group and between subgroups.

### Statistical Methods

We used SPSS version 26.0 statistical software to detect the distribution characteristics of measurement data, expressed as mean ± standard deviation or median and interquartile range, and count data expressed as rate (n%). A paired-sample *t*-test was used to compare with the rank sum test of two independent samples, and the characteristics were shown in a statistical graph. *p* < 0.05 is considered statistically significant.

## Results

### Patient Baseline Data

**FIGURE 1:**
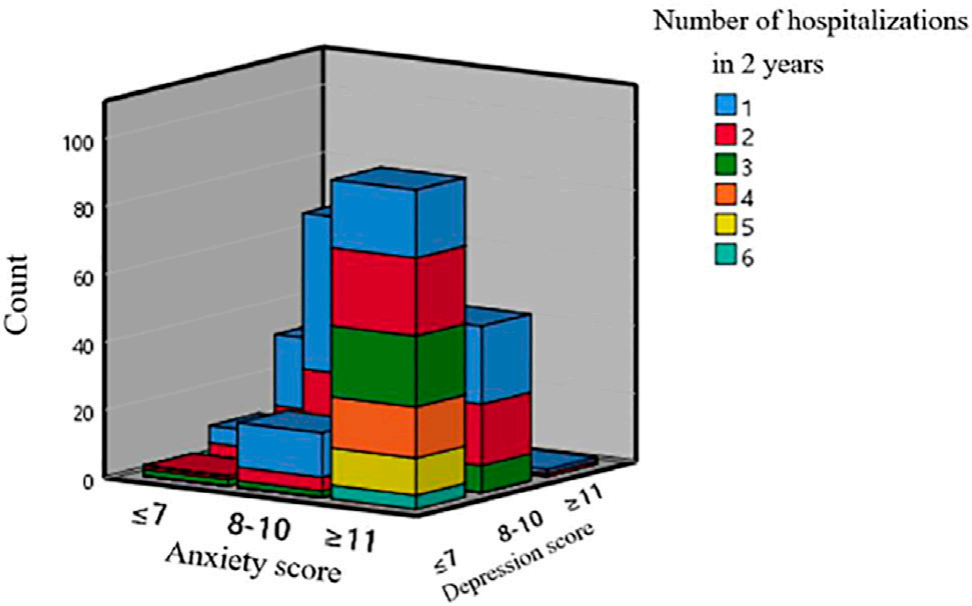
Three-dimensional bar graph of the number of hospitalizations and scores of AECOPD in a year.

There were 309 AECOPD patients admitted to the emergency department, 292/17 male/female, age 72.41 ± 9.266 (years old), body mass index 20.80 ± 3.282 (kg/㎡), and the number of hospitalizations was at least once within 2 years, and the number of hospitalizations was at most 6 times. Anxiety A score was 10.43 ± 2.741 (points), Depression D score was 9.83 ± 2.765 (points), Anxiety A–Depression D paired sample *t*-test revealed that the two are significantly positively correlated (r = 0.654, *p* = 0.000), and the paired difference has statistics learning significance (t = 4.560, *p* = 0.000). There were 45 cases (14.56%) in group A1, 119 cases (38.51%) in group A2, 145 cases (46.93%) in group A3, 58 cases (18.77%) in group D1, 135 cases (43.69%) in group D2, and 116 cases in group D3 (37.54%,Figure Figure1).

The influence of the number of hospitalizations within 2 years on anxiety of AECOPD admitted patients.

**FIGURE 2:**
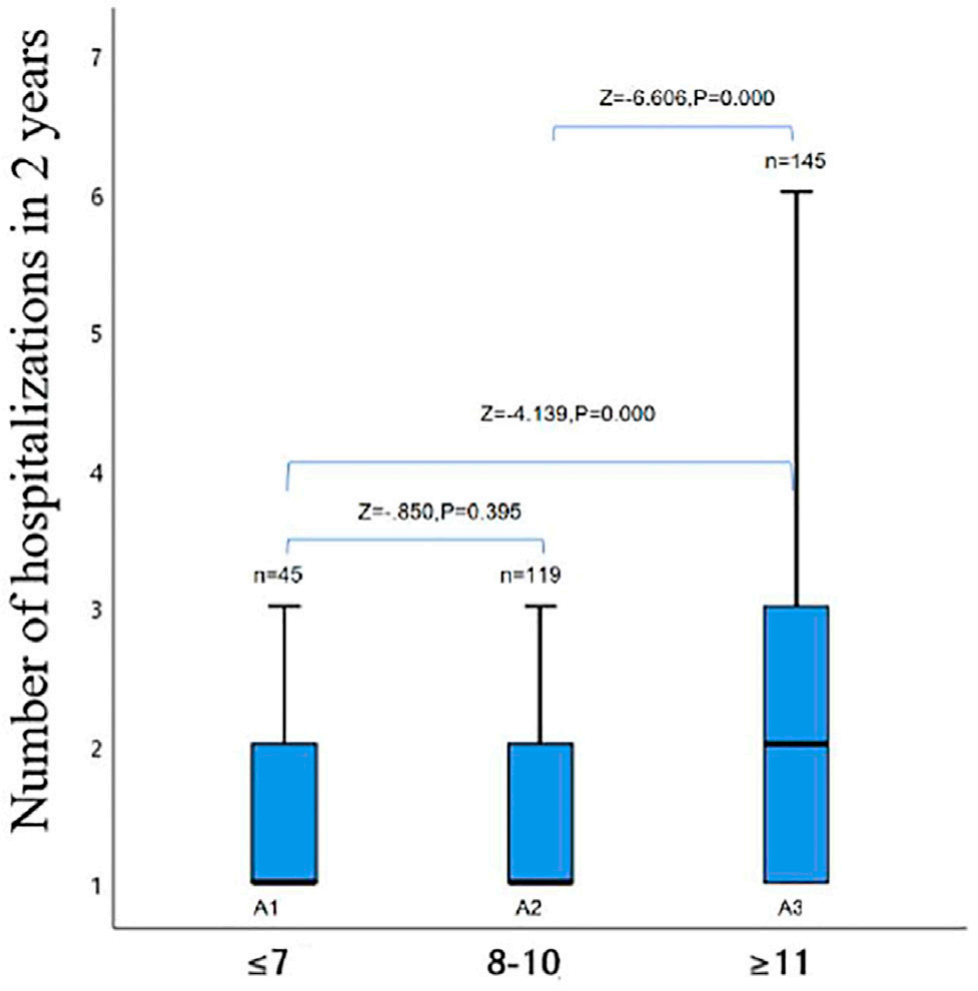
Intra-group comparison of the anxiety group.

The group analysis of the number of hospitalizations and depression scores of AECOPD admitted patients within 2 years showed that there was a statistical difference between the A1 group vs. the A3 group and the A2 group vs. the A3 group (*p* < 0.05), while the A1 group vs. the A2 group had no statistical difference (*p* > 0.05,Figure 2).

The influence of the number of hospitalizations in patients with AECOPD on depression within 2 years.

**FIGURE 3:**
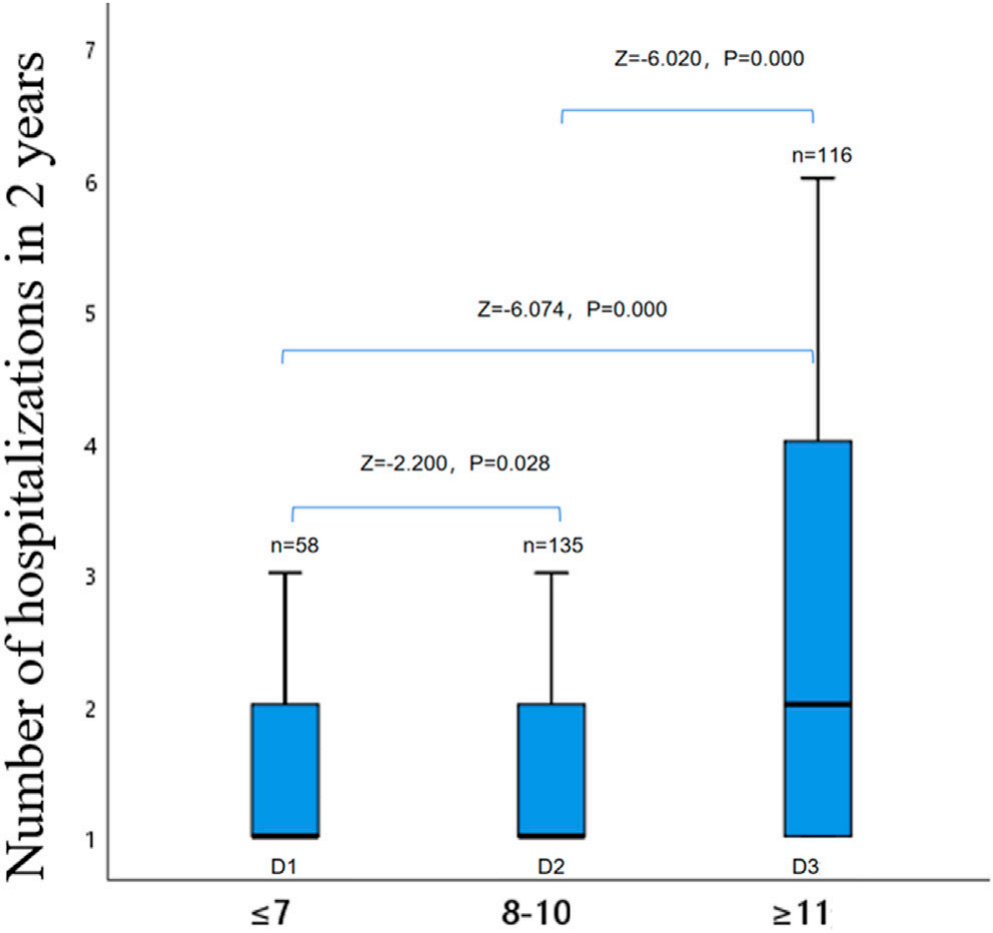
Comparison within the depression group.

The group analysis of the number of hospitalizations and depression scores of AECOPD admitted patients within 2 years showed that there were statistical differences between the D1 group and the D2 group, the D1 group and the D3 group, and the D2 group and the D3 group (*p* < 0.05, Figure 3).

The number of hospitalizations of patients admitted to AECOPD in the past 2 years was compared between the anxiety and depression subgroups.

**FIGURE 4:**
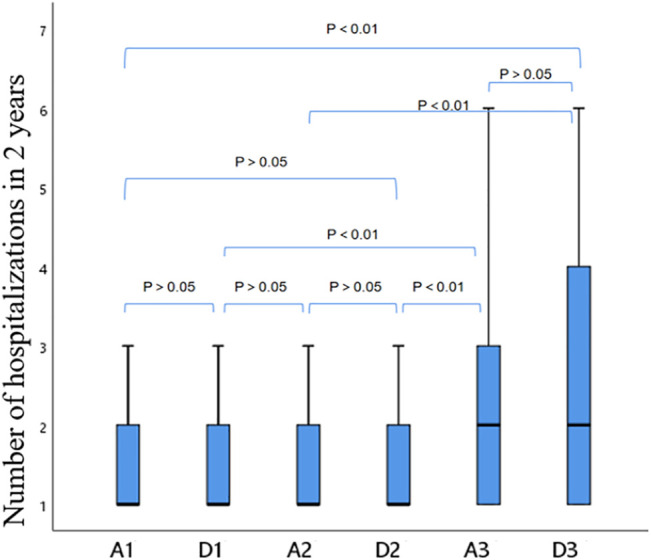
Comparison between anxiety subgroups and depression subgroups.

For the A1 group vs. the D1 group, the A1 group vs. the D2 group, the D1 group vs. the A2 group, and the A2 group vs. the D2 group, the 4 items are all undiagnosed anxiety and depression groups, and there is no statistical difference (*p* > 0.05); for the A3 group vs. the D3 group, it is the comparison between the confirmed groups, and there is no statistical difference (*p* > 0.05); for the A1 group vs. the D3 group, the A2 group vs. the D3 group, the D1 group vs. the A3 group, and the D2 group vs. the A3 group, these 4 items are the confirmed group. Compared with the non-diagnosed group, there was a significant statistical difference (*p* < 0.01, Figure 4).

## Discussion

Chronic obstructive pulmonary disease has more or less medical experience, including outpatient follow-up and hospitalization for acute exacerbations. The result of treatment is that it can be partially relieved, but it cannot be completely cured. It requires long-term medication control and respiratory function exercise, which can lead to fatigue, suspicion, anxiety, difficult behavior when it comes to cooperating with treatment, long-term sleep disorders, disease consumption, and deterioration of quality of life (Xu et al., 2010; Mohamadian et al., 2018; Huang et al., 2021; Pedrozo‐Pupo et al., 2021; Soler et al., 2021), which become more serious with the increase in age; the patient is adding a burden to the family and society, and smoking, coughing, and sputum can also lead to social avoidance. It is often considered that senile diseases are not paid enough attention, to a certain extent; due to lack of confidence in care and treatment, mental and emotional disorders are prone to appearing.

Anxiety and depression, as a comorbidity of many diseases, are a complication of chronic obstructive pulmonary disease, and the incidence is higher than other chronic diseases (García and González, 2021; Turnier et al., 2021; Wrzeciono et al., 2021). The two factors of dyspnea and anxiety are mutually stimulating, a vicious circle (Song, 2017). Anxiety and depression make breathing more difficult. In addition to chronic airway inflammation in COPD, there is also systemic inflammation, which can cause structural changes in the internal and external functions of the lung and affect neuroendocrine function (Salerno and Carone, 2011; Doyle et al., 2013; Lopez et al., 2013; Gordon et al., 2019). Long-term use of glucocorticoids in chronic obstructive pulmonary disease can cause the hippocampus to shrink, cause cognitive impairment, and aggravate anxiety and depression. Depression makes the body’s immunity weaker, complicated by infection and acute exacerbation of chronic obstructive pulmonary disease. The slow recovery of AECOPD can easily lead to anxiety and depression. The combination of chronic obstructive pulmonary disease and anxiety and depression interacts with each other and affects the quality of life of patients (Soler et al., 2021).

This study analyzed the relationship between the number of hospitalizations and anxiety and depression of AECOPD emergency patients in the past 2 years. Anxiety and depression, as two emotional factors, coexist and are positively correlated, and the average anxiety score of AECOPD patients is higher than the depression score. Anxiety subgroup analysis is as follows: when the anxiety score breaks the threshold of diagnosis, the corresponding number of hospitalizations within 2 years is high. Compared with the group with negative anxiety diagnosis and the suspicious group, there is no significant change in the number of hospitalizations. Different from the anxiety factor, the analysis within the depression subgroup is as follows: the higher the depression score, the higher the frequency of hospitalization within 2 years; the confirmed depression group>the depression suspicious group>the depression negative group. There is no statistical difference in the 2-year hospitalization frequency between the anxiety subgroup and the depression subgroup when the diagnostic threshold is not reached, and after the diagnosis threshold is exceeded, the two diagnosis subgroups of anxiety and depression have the same performance in hospitalization frequency. Comparison between the confirmed group and the non-diagnosed group reveals that the number of hospitalizations in the confirmed group was higher within 2 years, and the difference was significant. This may be because the anxiety in the acute exacerbation of COPD is more serious than the depression, and the increase in hospitalization frequency will make the patient anxious and depressed, which has a greater impact on anxiety, and the impact on depression is slowly and gradually aggravated, with the frequency of hospitalizations increasing from less to more. Depression scores range from mild to severe, and when the median hospitalization frequency is ≥ 2 times within 2 years, there is no statistical difference between anxiety and depression.

A prospective study showed that anxiety increases the number of symptom-related exacerbations and prolonged hospital stays, and depression may increase the risk of exacerbations and hospitalizations (Xu et al., 2010; Yohannes and Alexopoulos, 2014; von Leupoldt et al., 2011). Different from the prospective study, this study is a retrospective study of the number of hospitalizations in the past 2 years after admission. It is a single-factor analysis of the difference in anxiety and depression caused by the number of hospitalizations.

Anxiety and depression can increase the risk of hospitalization and the acute exacerbation of chronic obstructive pulmonary disease. Frequent hospitalization of AECOPD and anxiety and depression are mutually causal and a vicious circle. Cognitive behavioral therapy may relieve depression in COPD patients in a short time, but it takes longer to improve anxiety (Doyle et al., 2013; Lopez et al., 2013; Mohamadian et al., 2018; Gordon et al., 2019; Wrzeciono et al., 2021). Anxiety is more obvious in the acute phase, which can easily lead to poor treatment compliance and affect the efficacy. Multidisciplinary collaborative intervention may be more appropriate, and the stable period of COPD is more important for the early detection and investigation of depression.

In short, the increase in the frequency of AECOPD hospitalizations will aggravate anxiety and depression; anxiety and depression coexist in AECOPD patients. The appearance of depression is earlier and gradual. The early routine assessment of anxiety and depression in COPD patients should be carried out, for early intervention and interrupting the vicious circle, and patients admitted for AECOPD should give priority to the intervention and control of anxiety. However, this study also has certain limitations, for example, this study isolates anxiety and depression, and separately explores the effects of the two on the hospitalization frequency of patients. It does not further explore the effects of anxiety combined with depression, pure anxiety, and pure depression on the hospitalization frequency of patients. Second, although this study confirmed the negative impact of anxiety and depression on the frequency of hospitalization, it failed to comprehensively collect patient data for further multivariate analysis.

## Data Availability

The datasets presented in this study can be found in online repositories. The names of the repository/repositories and accession number(s) can be found in the article/supplementary material.
